# Effectiveness and safety of sotagliflozin adjuvant therapy for type 1 diabetes mellitus: A protocol for Systematic review and Meta-analysis

**DOI:** 10.1097/MD.0000000000016850

**Published:** 2019-08-16

**Authors:** Mao-Bing Chen, Rui-Jun Xu, Qi-Han Zheng, Xu-Wen Zheng, Hua Wang, Yun-Long Ding, Mao-Xing Yue

**Affiliations:** a Department of Emergency; b Department of Endocrinology; c Department of ICU, Wujin People Hospital, the Affiliated Hospital of Jiangsu University, Changzhou; d Department of Neurology, Jingjiang People Hospital, the Seventh Affiliated Hospital of Yangzhou University, Jingjiang, Jiangsu; e The people liberation Army 306 hospital, Beijing, PR China.

**Keywords:** meta-analysis, randomized controlled trials, SGLT-1 inhibitors, SGLT-2 inhibitors, sotagliflozin, type 1 diabetes mellitus

## Abstract

**Background:**

Type 1 Diabetes Mellitus (T1DM) has long required insulin treatment. Sotagliflflozin (SOTA), as a dual SGLT-1/2 inhibitor, has the potential to be the first oral antidiabetic drug (OAD) to be approved for T1DM in the US market. It is important to evaluate the effectiveness of SOTA for T1DM.

**Methods:**

Web of Science, PubMed datebase, Cochrane Library, Embase, Clinical Trials, and CNKI will be searched to identify randomized controlled trials (RCTs) exploring SOTA adjuvant therapy for T1DM. Strict screening and quality evaluation will be performed on the obtained literature independently by 2 researchers; outcome indexes will be extracted. The bias risk of the included studies will be evaluated based on Cochrane assessment tool. Meta-analysis will be performed on the data using Revman 5.3 software.

**Result:**

We will provide practical and targeted results assessing the efficacy and safety of SOTA for T1DM patients, to provide reference for clinical use of SOTA.

**Conclusion:**

The stronger evidence about the efficacy and safety of SOTA for T1DM patients will be provided for clinicians.

**Trial registration number:**

PROSPERO CRD42019133099.

## 1 Introduction

Type 1 diabetes mellitus, or insulin-dependent diabetes, is most common in children and adolescents, affecting millions of people worldwide.^[1]^ Owing to insufficient insulin production, patients have to use multiple daily injections (MDIs) of insulin or continuous subcutaneous insulin injection (CSII); otherwise, blood glucose cannot be well controlled.^[2]^ The incidence of T1DM is much lower than that of T2DM, but T1DM is more dangerous. Individuals with T1DM are prone to serious complications that can sometimes be life-threatening such as severe hypoglycemia, hypertonic coma, diabetic ketoacidosis, etc.^[3]^ For the treatment of T1DM, there are few noninsulin-assisted therapies; sodium-dependent glucose transporter-2 (SGLT-2) inhibitors are one of the popular topics in the research of diabetes drugs in recent years.^[4]^ SGLT-2 regulates blood glucose through the excretion function of the kidneys in addition to the metabolic pathway of glucose in the body by means of increasing the excretion of glucose by the kidneys.^[5]^ Sotagliflozin is a novel SGLT-1 / SGLT-2 dual inhibitor. Relying on its unique hypoglycemic mechanism, it reduces the absorption of glucose in the gastrointestinal tract by inhibiting SGLT-1 and increases the excretion of glucose by the kidneys by inhibiting SGLT-2.^[6]^ Studies have found that SOTA can not only treat T2DM but can also treat T1DM.^[7]^ Currently, SOTA has completed phase iii clinical trials (inTandem1, inTandem2, inTandem3).^[8]^ The purpose of this meta-analysis is to analyze the therapeutic effect and safety of SOTA on T1DM, thereby providing evidence for the treatment of T1DM by SOTA.

## 2 Methods

### 2.1 Design and registration

A meta-analysis will be conducted to evaluate the effectiveness and safety of SOTA adjuvant therapy for T1DM. This protocol has been registered on the international prospective register of systematic reviews (PROSPERO), registration number: CRD42019133099 (https://www.crd.york.ac.uk/PROSPERO). No ethical approval is required since this study used data that were already in the public domain.

### 2.2 Study selection

#### 2.2.1 Study type

Randomized Controlled Trials (RCTs).

#### 2.2.2 Study object

Type 1 diabetic patients who rely on insulin to control their glucose using MDIs or CSII to inject insulin, excluding individuals with other serious underlying diseases.

#### 2.2.3 Intervening measure

Patients received treatment for a period of time to stabilize their blood glucose and glycosylated hemoglobin (HbAlc) prior to the experiment. In the case of normal insulin therapy, SOTA tablets or placebo should be taken once a day.

#### 2.2.4 Outcome indicator

The following outcomes will be assessed compared with the effects of the placebo:

(1) differences in HbAlc,
(2) differences in the total daily insulin dose (TDD),
(3) differences in weight,
(4) differences in fasting blood glucose,
(5) differences in 2-hour postprandial blood glucose,
(6) differences in the rate of well-controlled diabetes (HbAlc < 7 after the end of the study, and no serious complications),
(7) differences in the probability of severe hypoglycemia,
(8) differences in the probability of diabetic ketoacidosis (DKA),
(9) differences in the probability of genital mycotic infections, and
(10) differences in the probability of urinary tract infections.

#### 2.2.5 Exclusion criteria

Literature whose data cannot be extracted or utilized, literature on animal experiments, literature reviews, etc.

### 2.3 Data sources and searches

**Figure 1:**
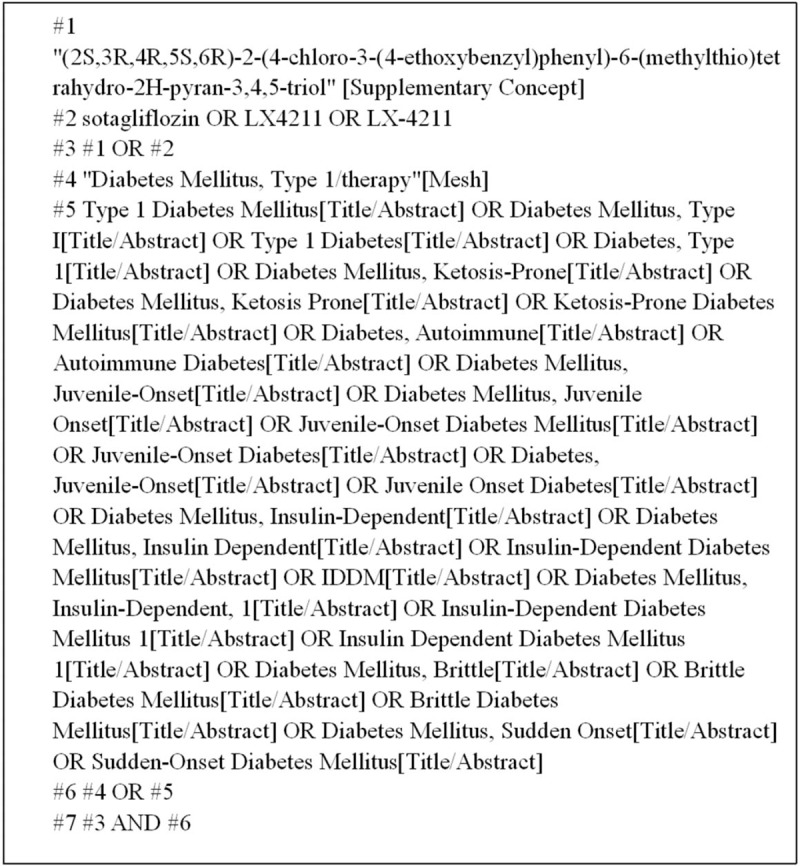
PubMed database retrieval strategy.

We will search English and Chinese language publications through July 2019 using the following databases: Web of Science, PubMed, Cochrane Library, Embase, Clinical Trails, and the China National Knowledge Infrastructure (CNKI). Search terms were “sotagliflozin”, “Type 1 Diabetes Mellitus”, “T1DM”, “LX4211” and so on. Here, we use the PubMed database as an example (see Fig. 1).

### 2.4 Study screening, data extraction and risk assessment of bias

Data will be collected independently by 2 researchers. The unqualified studies will be eliminated, and the qualified ones will be screened out after reading the title, abstract and full text. Then, the research data were extracted and checked, and disagreements were discussed or a decision was made by the author. The extracted data included the following:

1. basic information of the study, including title, author and year of publication;
2. characteristics of the included study, consisting of study duration, sample size of test group and control group, and intervention measures;
3. outcome indicators and data included; and
4. collection of risk assessment elements of bias.

The risk of bias in the included studies will be assessed by using the RCT bias risk assessment tool recommended in the Cochrane Handbook for Systematic Reviews of Interventions (5.1.0).

### 2.5 Statistical analysis

Revman 5.3 software will be used for the meta-analysis. The dichotomous variables will be relative risk (RR) as effect indicators, the continuous variables are expressed as mean difference (MD) as effect indicators, and the estimated value and 95% confidence interval (CI) will be included as effect analysis statistics. A heterogeneity test will be conducted with the results of each study. The fixed effect model will be used for analysis if there was no statistical heterogeneity between the results (I^2^ ≤ 50%). The sources of heterogeneity need to be analyzed if there was statistical heterogeneity between the results (I^2^ > 50%). After excluding the influence of obvious clinical heterogeneity, the random effect model will used for analysis. The significance level is set α = 0.05.

### 2.6 Subgroup analysis

Subgroups will be established based on difference of oral dose (400 mg/day, 200 mg/day).

### 2.7 Assessment of publication bias

If more than 10 articles are available for quantitative analysis, we will generate funnel plots to assess publication bias. A symmetrical distribution of funnel plot data indicates that there is no publication bias, otherwise, we will analyze the possible cause and give reasonable interpretation for asymmetric funnel plots.

### 2.8 Confidence in cumulative evidence

GRADE system will be used for assessing the quality of our evidence. According to the grading system, the level of evidence will be rated high, moderate, low, and very low.^[9]^

## 3 Discussion

SOTA is a new generation SGLT inhibitor that can act on both SGLT-1 and SGLT-2. SGLT-1 is mainly expressed in the small intestine and kidneys and is responsible for transporting glucose and galactose in the small intestine and reabsorbing glucose in the proximal convoluted tubules. SGLT-2 is specifically located in the proximal convoluted tubules of the kidney and is responsible for the renal reabsorption of glucose in the urine and is responsible for approximately 90% of glucose reabsorption.^[10]^

At present, the therapeutic effect of SOTA on TIDM is satisfactory.^[11]^ Literature supports that SOTA can reduce the HbAlc of T1DM patients, reduce the use of insulin does, and bring more patients to the standard.^[12,13]^ However, SOTA may cause kinds of serious adverse events.^[14]^ Among the common adverse reactions are urinary tract infection and genital infection. Meanwhile, SOTA may also increase the risk of diabetic ketoacidosis (DKA),^[15]^ although some studies believe that these data are not reliable.^[16]^ The comparison of benefits and losses in the treatment of TIDM by SOTA is still controversial.

This study will conduct a meta-analysis of related RCTs, and provide evidence on the efficacy and safety of SOTA in T1DM treatment, so as to better guide clinical practice.

## Author contributions

**Conceptualization:** Mao-Bing Chen, Rui-Jun Xu, Yun-Long Ding, Mao-Xing Yue.

**Data curation:** Mao-Bing Chen, Qi-Han Zheng, Xu-Wen Zheng, Hua Wang, Yun-Long Ding.

**Methodology:** Mao-Bing Chen, Hua Wang.

**Software:** Mao-Bing Chen, Xu-Wen Zheng.

**Supervision:** Mao-Bing Chen, Qi-Han Zheng.

**Writing – original draft:** Mao-Bing Chen, Rui-Jun Xu, Qi-Han Zheng, Xu-Wen Zheng, Hua Wang, Yun-long Ding, Mao-Xing Yue.

**Writing – review & editing:** Mao-Bing Chen.

Mao-bing Chen orcid: 0000-0001-5037-9870.

## References

[1] Alsahli M Gerich JE Hypoglycemia, chronic kidney disease, and diabetes mellitus. Mayo Clin Proc 2014;89:1564–71. 25305751 10.1016/j.mayocp.2014.07.013

[2] Little SA Speight J Leelarathna L Sustained reduction in severe hypoglycemia in adults with type 1 diabetes complicated by impaired awareness of hypoglycemia: two-year follow-up in the HypoCOMPaSS randomized clinical trial. Diabetes Care 2018;41:1600–7. 29661916 10.2337/dc17-2682

[3] Rossi MC Nicolucci A Ozzello A Impact of severe and symptomatic hypoglycemia on quality of life and fear of hypoglycemia in type 1 and type 2 diabetes. Results of the Hypos-1 observational study. Nutr Metab Cardiovasc Dis 2019;29:736–43. 31153746 10.1016/j.numecd.2019.04.009

[4] Miller K Miller EM Hot topics in primary care: role of the kidney and SGLT-2 inhibition in type 2 diabetes mellitus. J Fam Pract 2015;64 12 Suppl:S54–58. 26845015

[5] Deerochanawong C Pheng CS Matawaran BJ Use of SGLT-2 inhibitors in patients with type 2 diabetes mellitus and multiple cardiovascular risk factors: an Asian perspective and expert recommendations. Diabetes Obes Metab 2019. 10.1111/dom.13819 PMC6852284 31264765

[6] Markham A Keam SJ Sotagliflozin: first global approval. Drugs 2019;79:1023–9. 31172412 10.1007/s40265-019-01146-5

[7] Danne T Biester T Kordonouri O Combined SGLT1 and SGLT2 inhibitors and their role in diabetes care. Diabetes Technol Ther 2018;20 (S2):S269–77. 29916741 10.1089/dia.2018.0081

[8] McCrimmon RJ Henry RR SGLT inhibitor adjunct therapy in type 1 diabetes. Diabetologia 2018;61:2126–33. 30132030 10.1007/s00125-018-4671-6 PMC6133151

[9] Guyatt GH Oxman AD Vist GE GRADE: an emerging consensus on rating quality of evidence and strength of recommendations. BMJ 2008;336:924–6. 18436948 10.1136/bmj.39489.470347.AD PMC2335261

[10] Boeder S Edelman SV Sodium-glucose co-transporter inhibitors as adjunctive treatment to insulin in type 1 diabetes: a review of randomized controlled trials. Diabetes Obes Metab 2019;21 Suppl 2:62–77. 31081593 10.1111/dom.13749 PMC6899736

[11] Cefalo CMA Cinti F Moffa S Sotagliflozin, the first dual SGLT inhibitor: current outlook and perspectives. Cardiovasc Diabetol 2019;18:20. 30819210 10.1186/s12933-019-0828-y PMC6393994

[12] Buse JB Garg SK Rosenstock J Sotagliflozin in combination with optimized insulin therapy in adults with type 1 diabetes: the North American inTandem1 study. Diabetes Care 2018;41:1970–80. 29937430 10.2337/dc18-0343 PMC6105319

[13] Danne T Cariou B Buse JB Improved time in range and glycemic variability with sotagliflozin in combination with insulin in adults with type 1 diabetes: a pooled analysis of 24-week continuous glucose monitoring data from the inTandem program. Diabetes Care 2019;42:919–30. 30833371 10.2337/dc18-2149 PMC6905498

[14] He YL Haynes W Meyers CD The effects of licogliflozin, a dual SGLT1/2 inhibitor, on body weight in obese patients with or without diabetes. Diabetes Obes Metab 2019;21:1311–21. 30724002 10.1111/dom.13654

[15] Goldenberg RM Gilbert JD Hramiak IM Sodium-glucose co-transporter inhibitors, their role in type 1 diabetes treatment and a risk mitigation strategy for preventing diabetic ketoacidosis: the STOP DKA Protocol. Diabetes Obes Metab 2019. 10.1111/dom.13811 31183975

[16] Prattichizzo F De Nigris V Micheloni S Increases in circulating levels of ketone bodies and cardiovascular protection with SGLT2 inhibitors: is low-grade inflammation the neglected component? Diabetes Obes Metab 2018;20:2515–22. 30073768 10.1111/dom.13488

